# Supplementary material for: Independent phase modulation for quadruplex polarization channels enabled by chirality-assisted geometric-phase metasurfaces
Source: Nat Commun. 2020 Aug 21;11:4186. doi: 10.1038/s41467-020-17773-6 (PMC7442839; doi:10.1038/s41467-020-17773-6)
Supplement: Supplementary file 1 — Supplementary Information [file 41467_2020_17773_MOESM1_ESM.pdf]

Supplementary Information for

Independent phase modulation for quadruplex polarization channels enabled by chirality-assisted geometric-phase metasurfaces

Yuan et al.

Supplementary Note 1: Mechanism of the proposed meta-atom

Referring to the equivalent circuit of filter in microwave region, the basic element block is composed of five metallic layers and four dielectric substrate layers as shown in Supplementary Figure 1a. The odd-numbered metallic layers are occupied by rectangular metallic patches, acting as capacitive component (C_n) in the equivalent band-pass filter circuit. Three parallel gaps are implemented in each rectangular patch for miniaturization purpose. The even-numbered layers constituted by metallic grids with centered circular apertures play the role of equivalent inductor (L_n), guaranteeing polarization insensitivity during the rotation of the patch layers. The dielectric substrates with similar thickness are regarded as transmission lines with impedance Z_n. Each adjacent capacitive and inductive layers can be regarded as an L-C resonant element in the equivalent circuit, and the whole block enables 4th order resonances to generate a broad frequency bandwidth with high transmission coefficient under linearly polarized illuminations and whole 2π phase coverage necessary for arbitrary CP wave stimulation and modulation based on synthesizing three different phase schemes.

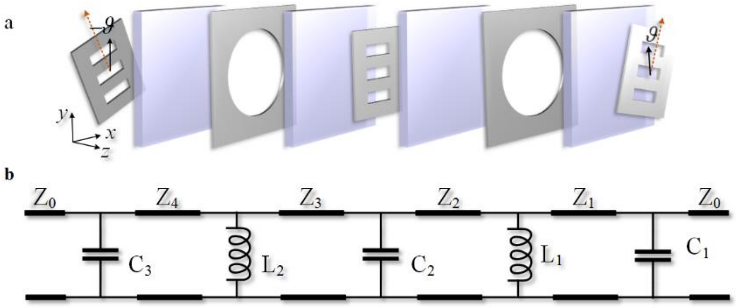

Supplementary Figure 1. Schematic of proposed meta-atom. **a** Topological layout of meta-atom corresponding to schematic in Supplementary Figure 1a of main text. **b** Equivalent 4th order resonance circuit model of proposed meta-atom.

Furthermore, the basic electromagnetic characteristics of this proposed meta-atom and its equivalent network is discussed in this part.

1. *Reciprocity*. Since the meta-atom design proposed in this paper is symmetric in structure and totally passive (without any active component), all of the proposed meta-atoms are reciprocal. It means that there exist $S_{12}^{xy}=S_{21}^{yx}$, $S_{12}^{yx}=S_{21}^{xy}$ in the general scattering matrix, where the first (second) subscript presents for the region of incident (scattered) field, and the first (second) superscript shows the polarization state of incident (scattered) wave. Additionally, the reciprocity is verified by simulations as shown in Supplementary Figure 2, where the scattering responses of transmission coefficients are exhibited. Supplementary Figure 2a and 2b show the amplitude and phase responses of meta-atom with

p_x = 4.8 mm, p_y = 5.7 mm, ϑ = 0 and θ = 0, Supplementary Figure 2c and 2d exhibit the amplitude and phase responses of meta-atom with p_x = 4.8 mm, p_y = 5.7 mm, $\vartheta = 30^{\circ}$ and θ = 0, Supplementary Figure 2e and 2f present the amplitude and phase responses of meta-atom with p_x = 4.8 mm, p_y = 5.7 mm, $\vartheta = 30^{\circ}$ and $\theta = 30^{\circ}$. It can be seen that the transmission curves S_{12}^{xy} and S_{21}^{yx} of all these three meta-atoms are in agreement, while S_{12}^{yx} and S_{21}^{xy} are also equal, which successfully verify the reciprocity of proposed structure. In fact, in our proposed scheme for full transmission phase manipulation, we just consider one propagation direction (from port 1 to port 2) of the electromagnetic wave.

2. *Lossless.* In microwave region, metal can be regarded as perfect electric conductor and the loss tangent of dielectric materials is usually in the order of 10^{-3}. Thus, the system can be supposed as a lossless system in the analyses and simulations. Practically, if the system is proposed with lossy media, the equivalent scattering matrix would not be unitary and the efficiency of the system would be further affected and reduced. Since our scheme only works on the phase modulation, the change of amplitude would not affect the application of the scheme and it is still effective.

3. *Matching.* The ideal model is supposed to be matched to free space, which is convenient for calculating the phase-modulation process. However, since there is inevitable reflection within the design process of metasurface (or meta-atom) structures, the practical system would be mismatched. During the construction of metasurfaces, the phase responses of meta-atoms have to approach discrete spatial phase distribution, for example required for deflection or OAM generation. To achieve the phase response as accurate as possible, there would be some optimization process of the meta-atom's phase response and the amplitude response would inevitably be sacrificed. On the other hand, the interior and exterior rotation in meta-atom structure would also introduce mismatching in the system, resulting in the increase of reflection components. As it can be observed from Supplementary Figure 2a, 2c and 2e, when the meta-atom is imposed with the interior and exterior rotations, the amplitude of S^{xx} and S^{yy} are gradually decreased. So, equivalent network of the meta-atom is considered mismatched to free space.

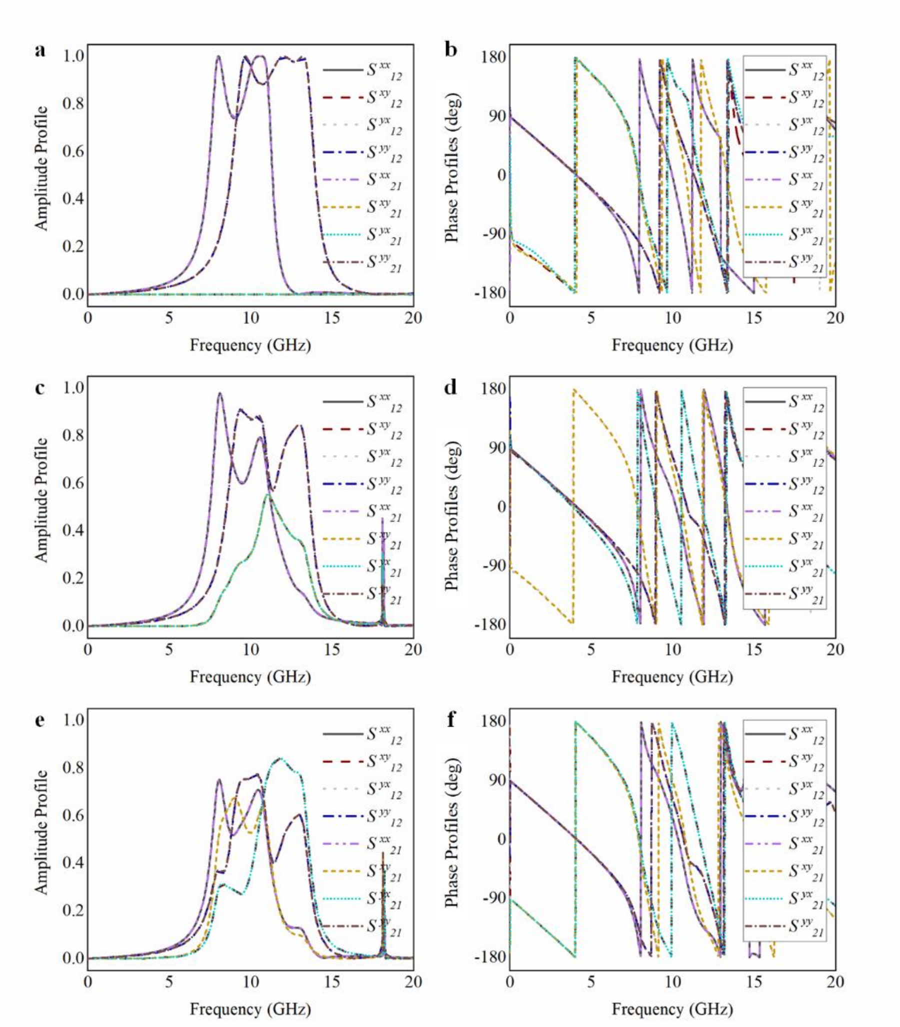

Supplementary Figure 2. Simulated scattering responses of three proposed meta-atoms with different parameter dimensions, where **a**, **c**, **e** show the amplitude responses and **b**, **d**, **f** exhibit the phase profiles.

Supplementary Note 2: Analysis of chirality-assisted phase introduced by the proposed meta-atom

The chirality-assisted phase factor is introduced by interior rotation between adjacent patch layers of the meta-atom, which is simply achieved by rotating each single patch layer with different angles α_1, α_2 and α_3. Here we suppose that each patch layer is considered as a birefringent wave plate with four-fold symmetric structure, and it can be equivalent to $T_0 = \begin{bmatrix} t_{0\mathrm{xx}} & 0 \\ 0 & t_{0\mathrm{yy}} \end{bmatrix}$ (the linear coefficients of single-layer patch is supposed as $t_{0\mathrm{xx}} \neq t_{0\mathrm{yx}}$ and $t_{0\mathrm{xy}} = t_{0\mathrm{yx}} = 0$). Thus, the total transmission matrix $\boldsymbol{T}_{\mathrm{cas}}$, which is considered as a cascaded model composing three independent single-layer patched with rotation angles α_1, α_2 and α_3, can be calculated and described as:

$$
\begin{aligned}
T_{\mathrm{cas}} &= T_1(\alpha_1)\cdot T_2(\alpha_2)\cdot T_3(\alpha_3) \\
&= \{\frac{1}{2}[t_{0\mathrm{xx}}+t_{0\mathrm{yy}}]\cdot I+\frac{1}{2}[t_{0\mathrm{xx}}-t_{0\mathrm{yy}}]\cdot(\mathrm{e}^{\mathrm{i}\cdot 2\alpha_1}\cdot\hat{\sigma}_{\mathrm{R}}+\mathrm{e}^{-\mathrm{i}\cdot 2\alpha_1}\cdot\hat{\sigma}_{\mathrm{L}})\} \\
&\cdot\{\frac{1}{2}[t_{0\mathrm{xx}}+t_{0\mathrm{yy}}]\cdot I+\frac{1}{2}[t_{0\mathrm{xx}}-t_{0\mathrm{yy}}]\cdot(\mathrm{e}^{\mathrm{i}\cdot 2\alpha_2}\cdot\hat{\sigma}_{\mathrm{R}}+\mathrm{e}^{-\mathrm{i}\cdot 2\alpha_2}\cdot\hat{\sigma}_{\mathrm{L}})\} \\
&\cdot\{\frac{1}{2}[t_{0\mathrm{xx}}+t_{0\mathrm{yy}}]\cdot I+\frac{1}{2}[t_{0\mathrm{xx}}-t_{0\mathrm{yy}}]\cdot(\mathrm{e}^{\mathrm{i}\cdot 2\alpha_3}\cdot\hat{\sigma}_{\mathrm{R}}+\mathrm{e}^{-\mathrm{i}\cdot 2\alpha_3}\cdot\hat{\sigma}_{\mathrm{L}})\}
\end{aligned}
\tag{S1}
$$

In order to simplify the cascaded Jones matrix conveniently, here, the rotation angles of each patch layer is set as $\alpha_1 = -\vartheta$, $\alpha_2 = 0$, and $\alpha_3 = \vartheta$, and the cascaded transmission expression can be obtained as:

$$
\begin{aligned}
T_{\mathrm{cas}} &= \frac{1}{8}(t_{0\mathrm{xx}}+t_{0\mathrm{yy}})^3\cdot I \\
&+\frac{1}{8}[(t_{0\mathrm{xx}}+t_{0\mathrm{yy}})\cdot(t_{0\mathrm{xx}}-t_{0\mathrm{yy}})^2\cdot(2\mathrm{e}^{\mathrm{i}\cdot 2\vartheta}+\mathrm{e}^{\mathrm{i}\cdot 4\vartheta})]\cdot|\mathrm{L}\rangle\langle\mathrm{L}| \\
&+\frac{1}{8}[(t_{0\mathrm{xx}}+t_{0\mathrm{yy}})(t_{0\mathrm{xx}}-t_{0\mathrm{yy}})^2\cdot(2\mathrm{e}^{-\mathrm{i}\cdot 2\vartheta}+\mathrm{e}^{-\mathrm{i}\cdot 4\vartheta})]\cdot|\mathrm{R}\rangle\langle\mathrm{R}| \\
&+\frac{1}{8}[(t_{0\mathrm{xx}}-t_{0\mathrm{yy}})^3+(t_{0\mathrm{xx}}+t_{0\mathrm{yy}})^2\cdot(t_{0\mathrm{xx}}-t_{0\mathrm{yy}})\cdot(1+\mathrm{e}^{-\mathrm{i}\cdot 2\vartheta}+\mathrm{e}^{\mathrm{i}\cdot 2\vartheta})]\cdot|\mathrm{L}\rangle\langle\mathrm{R}| \\
&+\frac{1}{8}[(t_{0\mathrm{xx}}-t_{0\mathrm{yy}})^3+(t_{0\mathrm{xx}}+t_{0\mathrm{yy}})^2\cdot(t_{0\mathrm{xx}}-t_{0\mathrm{yy}})\cdot(1+\mathrm{e}^{\mathrm{i}\cdot 2\vartheta}+\mathrm{e}^{-\mathrm{i}\cdot 2\vartheta})]\cdot|\mathrm{R}\rangle\langle\mathrm{L}|
\end{aligned}
\tag{S2}
$$

Based on Eq. (S2), the Jones matrix of the meta-atom, which is set with the specific interior rotation and no exterior rotation, can be extracted and illustrated by T_1:

$$T_1(\vartheta) = \begin{bmatrix} T_{1\mathrm{LL}} & T_{1\mathrm{LR}} \\ T_{1\mathrm{RL}} & T_{1\mathrm{RR}} \end{bmatrix}$$

$$= \frac{1}{8}\begin{bmatrix} (t_{0\mathrm{xx}}+t_{0\mathrm{yy}})^3 + (t_{0\mathrm{xx}}+t_{0\mathrm{yy}})\cdot(t_{0\mathrm{xx}}-t_{0\mathrm{yy}})^2\cdot(2\mathrm{e}^{\mathrm{i}\cdot 2\vartheta}+\mathrm{e}^{\mathrm{i}\cdot 4\vartheta}) & (t_{0\mathrm{xx}}-t_{0\mathrm{yy}})^3 + 2\cdot(t_{0\mathrm{xx}}+t_{0\mathrm{yy}})^2\cdot(t_{0\mathrm{xx}}-t_{0\mathrm{yy}})\cdot\cos 2\vartheta \\ (t_{0\mathrm{xx}}-t_{0\mathrm{yy}})^3 + 2\cdot(t_{0\mathrm{xx}}+t_{0\mathrm{yy}})^2\cdot(t_{0\mathrm{xx}}-t_{0\mathrm{yy}})\cdot\cos 2\vartheta & (t_{0\mathrm{xx}}+t_{0\mathrm{yy}})^3 + (t_{0\mathrm{xx}}+t_{0\mathrm{yy}})(t_{0\mathrm{xx}}-t_{0\mathrm{yy}})^2\cdot(2\mathrm{e}^{-\mathrm{i}\cdot 2\vartheta}+\mathrm{e}^{-\mathrm{i}\cdot 4\vartheta}) \end{bmatrix} \tag{S3}$$

In order to calculate the output phase patterns in four transmission coefficients in T_1, here it is supposed that the linear transmission amplitude for each ideal birefringent wave plate model is regarded as 1 ($|t_{0\mathrm{xx}}| = |t_{0\mathrm{yy}}| = 1$). The phase difference and phase combination between the two linear orthogonal directions are defined as $\Delta\varphi = \varphi_{0\mathrm{xx}} - \varphi_{0\mathrm{yy}}$ and $\Sigma\varphi = \varphi_{0\mathrm{xx}} + \varphi_{0\mathrm{yy}}$. For the further simplification and calculation of Jones matrix of equivalent meta-atom with interior rotation and no exterior rotation, the following two relationships can be obtained:

$$\frac{1}{2}(t_{0\mathrm{xx}}+t_{0\mathrm{yy}}) = \cos\frac{\Delta\varphi}{2}\cdot\mathrm{e}^{\mathrm{i}\cdot\frac{\Sigma\varphi}{2}} \tag{S4a}$$

$$\frac{1}{2}(t_{0\mathrm{xx}}+t_{0\mathrm{yy}}) = \mathrm{i}\cdot\sin\frac{\Delta\varphi}{2}\cdot\mathrm{e}^{\mathrm{i}\cdot\frac{\Sigma\varphi}{2}} \tag{S4b}$$

By substituting Eq. (S4) into Eq. (S3), phase patterns of four transmission channels, introduced by the chirality responses of interior rotation angle ϑ, can be deduced as:

$$\varphi_{1\mathrm{LL}} = \arg[\cos^3\frac{\Delta\varphi}{2}\cdot\mathrm{e}^{\mathrm{i}\cdot\frac{3}{2}\Sigma\varphi} + (\cos\frac{\Delta\varphi}{2}\cdot\sin^2\frac{\Delta\varphi}{2}\cdot\mathrm{e}^{\mathrm{i}\cdot\frac{3}{2}\Sigma\varphi+\pi/2})\cdot(2\mathrm{e}^{\mathrm{i}\cdot 2\vartheta}+\mathrm{e}^{\mathrm{i}\cdot 4\vartheta})] \tag{S5a}$$

$$\varphi_{1\mathrm{RR}} = \arg[\cos^3\frac{\Delta\varphi}{2}\cdot\mathrm{e}^{\mathrm{i}\cdot\frac{3}{2}\Sigma\varphi} + (\cos\frac{\Delta\varphi}{2}\cdot\sin^2\frac{\Delta\varphi}{2}\cdot\mathrm{e}^{\mathrm{i}\cdot\frac{3}{2}\Sigma\varphi+\pi/2})\cdot(2\mathrm{e}^{-\mathrm{i}\cdot 2\vartheta}+\mathrm{e}^{-\mathrm{i}\cdot 4\vartheta})] \tag{S5b}$$

$$\varphi_{1\mathrm{LR}} = \varphi_{1\mathrm{RL}} = \arg[(2\cdot\cos^2\frac{\Delta\varphi}{2}\cdot\sin\frac{\Delta\varphi}{2}\cdot\cos 2\vartheta\text{-}\sin^3\frac{\Delta\varphi}{2})\mathrm{e}^{\mathrm{i}\cdot\frac{3}{2}\Sigma\varphi+\pi/2}] \tag{S5c}$$

Eq. (S5) expresses the chirality-assisted phase functions of four CP channels transmission coefficients. It can be seen that the phase patterns of two co-polarized channels L-L and R-R have been decoupled with different phase pattern by introducing interior rotation angle ϑ, while the cross-polarized output phase in channel L-R and R-L are still maintained in same state. It is indicated that the interior rotation angle ϑ would just produce decoupling influence in the co-polarized channels and would have no effect on the cross-polarized output fields. It should be noted that in this part, Eq. (S5) is established on the idealized assumption that the three patch layers are considered as perfectly independent ideal wave plates. However, practically in this work, the three patch layers are metallic elements in the equivalent filter circuit as analyzed in Supplementary Note 1, meaning that the coupling between each layer would unavoidably affect the output final phase. Therefore, the phase in four CP channels of each meta-atom

for metadevices construction (as illustrated in Fig. 3b in main text), are finally fixed with full wave simulation and optimization processing, which can effectively counteract the influence produced by metallic-layer coupling.

Moreover, as for the proposed meta-atom imposed with both interior and exterior rotation, the equivalent Jones matrix is expressed as $T=\begin{bmatrix} T_{\mathrm{LL}} & T_{\mathrm{LR}} \\ T_{\mathrm{RL}} & T_{\mathrm{RR}} \end{bmatrix}$, which is further calculated based on T_1. It is known that the exterior rotation provides the geometric phase and the exterior rotation angle is set as θ. The derivation process can be described as follows:

$$
\begin{aligned}
T(\vartheta,\theta) &= M(\theta)^T \cdot T_1(\vartheta)\cdot M(\theta) \\
&= \begin{bmatrix} \cos\theta & -\sin\theta \\ \sin\theta & \cos\theta \end{bmatrix} \cdot \begin{bmatrix} T_{\mathrm{1LL}} & T_{\mathrm{1LR}} \\ T_{\mathrm{1RL}} & T_{\mathrm{1RR}} \end{bmatrix} \cdot \begin{bmatrix} \cos\theta & \sin\theta \\ -\sin\theta & \cos\theta \end{bmatrix} \\
&= \begin{bmatrix} T_{\mathrm{1LL}}\cdot\cos^2\theta + T_{\mathrm{1RR}}\cdot\sin^2\theta - (T_{\mathrm{1LR}}+T_{\mathrm{1RL}})\cdot\sin\theta\cdot\cos\theta & (T_{\mathrm{1RR}}-T_{\mathrm{1LL}})\cdot\sin\theta\cdot\cos\theta + T_{\mathrm{1LR}}\cdot\cos^2\theta - T_{\mathrm{1RL}}\cdot\sin^2\theta \\ (T_{\mathrm{1RR}}-T_{\mathrm{1LL}})\cdot\sin\theta\cdot\cos\theta - T_{\mathrm{1LR}}\cdot\sin^2\theta + T_{\mathrm{1RL}}\cdot\cos^2\theta & T_{\mathrm{1LL}}\cdot\sin^2\theta + T_{\mathrm{1RR}}\cdot\cos^2\theta + (T_{\mathrm{1LR}}+T_{\mathrm{1RL}})\cdot\sin\theta\cdot\cos\theta \end{bmatrix} \\
&= \begin{bmatrix} A & B \\ C & D \end{bmatrix}
\end{aligned}
\tag{S6}
$$

where $\mathbf{M}(\theta)=\begin{bmatrix} \cos\theta & \sin\theta \\ -\sin\theta & \cos\theta \end{bmatrix}$ is the rotation matrix. Eq. (S6) expresses the general form of Jones matrix. Four elements A, B, C, D are independent from each other and represent for different transmission characteristics in the four channels, which are described as:

$$A=\frac{1}{2}(T_{\mathrm{1LL}}+T_{\mathrm{1RR}})+\frac{1}{4}[(T_{\mathrm{1LL}}-T_{\mathrm{1RR}})+\mathrm{i}\cdot(T_{\mathrm{1LR}}+T_{\mathrm{1RL}})]\cdot \mathrm{e}^{\mathrm{i}\cdot 2\theta}+\frac{1}{4}[(T_{\mathrm{1LL}}-T_{\mathrm{1RR}})-\mathrm{i}\cdot(T_{\mathrm{1LR}}+T_{\mathrm{1RL}})]\cdot \mathrm{e}^{-\mathrm{i}\cdot 2\theta} \tag{S7a}$$

$$B=\frac{1}{2}(T_{\mathrm{1LR}}-T_{\mathrm{1RL}})+\frac{1}{4}[(T_{\mathrm{1LR}}+T_{\mathrm{1RL}})-\mathrm{i}\cdot(T_{\mathrm{1LL}}-T_{\mathrm{1RR}})]\cdot \mathrm{e}^{\mathrm{i}\cdot 2\theta}+\frac{1}{4}[(T_{\mathrm{1LR}}+T_{\mathrm{1RL}})+\mathrm{i}\cdot(T_{\mathrm{1LL}}-T_{\mathrm{1RR}})]\cdot e^{-\mathrm{i}\cdot 2\theta} \tag{S7b}$$

$$C=\frac{1}{2}(T_{\mathrm{1RL}}-T_{\mathrm{1LR}})+\frac{1}{4}[(T_{\mathrm{1LR}}+T_{\mathrm{1RL}})-\mathrm{i}\cdot(T_{\mathrm{1LL}}-T_{\mathrm{1RR}})]\cdot e^{\mathrm{i}\cdot 2\theta}+\frac{1}{4}[(T_{\mathrm{1LR}}+T_{\mathrm{1RL}})+\mathrm{i}\cdot(T_{\mathrm{1LL}}-T_{\mathrm{1RR}})]\cdot e^{-\mathrm{i}\cdot 2\theta} \tag{S7c}$$

$$D=\frac{1}{2}(T_{\mathrm{1LL}}+T_{\mathrm{1RR}})+\frac{1}{4}[(T_{\mathrm{1RR}}-T_{\mathrm{1LL}})-\mathrm{i}\cdot(T_{\mathrm{1LR}}+T_{\mathrm{1RL}})]\cdot \mathrm{e}^{\mathrm{i}\cdot 2\theta}+\frac{1}{4}[(T_{\mathrm{1RR}}-T_{\mathrm{1LL}})+\mathrm{i}\cdot(T_{\mathrm{1LR}}+T_{\mathrm{1RL}})]\cdot \mathrm{e}^{-\mathrm{i}\cdot 2\theta} \tag{S7d}$$

From Eq. (S5) we can note that the two cross-polarized phase responses are similar to each other when there is only interior rotation in the meta-atom structure, leading to $\varphi_{\mathrm{1LR}}=\varphi_{\mathrm{1RL}}$. Through substituting Eq. (S3) into Eq. (S6-S7), the meta-atom with both interior and exterior rotations can be equivalent to Jones matrix of meta-atom as described:

$$A(t_{0\mathrm{xx}},t_{0\mathrm{yy}},\vartheta,\theta)=\frac{1}{8}[(t_{0\mathrm{xx}}+t_{0\mathrm{yy}})^3+(t_{0\mathrm{xx}}+t_{0\mathrm{yy}})\cdot(t_{0\mathrm{xx}}-t_{0\mathrm{yy}})^2\cdot(2\cos 2\vartheta+\cos 4\vartheta)]$$
$$+\frac{\mathrm{i}}{8}[(t_{0\mathrm{xx}}+t_{0\mathrm{yy}})\cdot(t_{0\mathrm{xx}}-t_{0\mathrm{yy}})^2\cdot(2\sin 2\vartheta+\sin 4\vartheta)]\cos 2\theta \qquad \text{(S8a)}$$
$$+\frac{1}{8}[(t_{0\mathrm{xx}}-t_{0\mathrm{yy}})^3+2\cdot(t_{0\mathrm{xx}}+t_{0\mathrm{yy}})^2\cdot(t_{0\mathrm{xx}}-t_{0\mathrm{yy}})\cdot\cos 2\vartheta]\sin 2\theta$$

$$B(t_{0\mathrm{xx}},t_{0\mathrm{yy}},\vartheta,\theta)=\frac{\mathrm{i}}{8}[(t_{0\mathrm{xx}}+t_{0\mathrm{yy}})\cdot(t_{0\mathrm{xx}}-t_{0\mathrm{yy}})^2\cdot(2\sin 2\vartheta+\sin 4\vartheta)]\cdot\sin 2\theta$$
$$+\frac{1}{8}[(t_{0\mathrm{xx}}-t_{0\mathrm{yy}})^3+2\cdot(t_{0\mathrm{xx}}+t_{0\mathrm{yy}})^2\cdot(t_{0\mathrm{xx}}-t_{0\mathrm{yy}})\cdot\cos 2\vartheta]\cdot\cos 2\theta \qquad \text{(S8b)}$$

$$C(t_{0\mathrm{xx}},t_{0\mathrm{yy}},\vartheta,\theta)=\frac{\mathrm{i}}{8}[(t_{0\mathrm{xx}}+t_{0\mathrm{yy}})\cdot(t_{0\mathrm{xx}}-t_{0\mathrm{yy}})^2\cdot(2\sin 2\vartheta+\sin 4\vartheta)]\cdot\sin 2\theta$$
$$+\frac{1}{8}[(t_{0\mathrm{xx}}-t_{0\mathrm{yy}})^3+2\cdot(t_{0\mathrm{xx}}+t_{0\mathrm{yy}})^2\cdot(t_{0\mathrm{xx}}-t_{0\mathrm{yy}})\cdot\cos 2\vartheta]\cdot\cos 2\theta \qquad \text{(S8c)}$$

$$D(t_{0\mathrm{xx}},t_{0\mathrm{yy}},\vartheta,\theta)=\frac{1}{8}[(t_{0\mathrm{xx}}+t_{0\mathrm{yy}})^3+(t_{0\mathrm{xx}}+t_{0\mathrm{yy}})\cdot(t_{0\mathrm{xx}}-t_{0\mathrm{yy}})^2\cdot(2\cos 2\vartheta+\cos 4\vartheta)]$$
$$-\frac{\mathrm{i}}{8}[(t_{0\mathrm{xx}}+t_{0\mathrm{yy}})\cdot(t_{0\mathrm{xx}}-t_{0\mathrm{yy}})^2\cdot(2\sin 2\vartheta+\sin 4\vartheta)]\cos 2\theta \qquad \text{(S8d)}$$
$$+\frac{1}{8}[(t_{0\mathrm{xx}}-t_{0\mathrm{yy}})^3+2\cdot(t_{0\mathrm{xx}}+t_{0\mathrm{yy}})^2\cdot(t_{0\mathrm{xx}}-t_{0\mathrm{yy}})\cdot\cos 2\vartheta]\sin 2\theta$$

where $t_{0\mathrm{xx}}$ and $t_{0\mathrm{yy}}$ represent the linear transmission coefficients of birefringent single-layer patch, ϑ is the interior rotation angle provided by three independent patch layers, and θ shows the exterior angle introduced by rotating the whole meta-atom with all three patch layers.

Supplementary Note 3: Derivation of basic method for constructing meta-devices

It is supposed that the orthogonal circularly polarized incident waves can be described as $\vec{E}_{\text{in}} = |\vec{\text{L}}\rangle = \begin{bmatrix} 1 \\ \text{i} \end{bmatrix}$ (or $\vec{E}_{\text{in}} = |\vec{\text{R}}\rangle = \begin{bmatrix} 1 \\ \text{-i} \end{bmatrix}$). After passing through the proposed metasurface structure, which can separately manipulate the wavefronts of all four CP channels L-L, L-R, R-L and R-R, the output components are imposed with four different phase distributions $F_{\text{LL}}(x,y)$, $F_{\text{LR}}(x,y)$, $F_{\text{RL}}(x,y)$, and $F_{\text{RR}}(x,y)$. This process can be expressed as:

$$\vec{E}_{\text{out}}^{\text{L,in}} = T \cdot |\text{L}\rangle = \text{e}^{\text{i}\cdot F_{\text{LL}}(x,y)} \cdot |\text{L}\rangle + \text{e}^{\text{i}\cdot F_{\text{LR}}(x,y)} \cdot |\text{R}\rangle \tag{S9a}$$

$$\vec{E}_{\text{out}}^{\text{R,in}} = T \cdot |\text{R}\rangle = \text{e}^{\text{i}\cdot F_{\text{RL}}(x,y)} \cdot |\text{L}\rangle + \text{e}^{\text{i}\cdot F_{\text{RR}}(x,y)} \cdot |\text{R}\rangle \tag{S9b}$$

Upon integrating Eq. (S9), we can obtain the required Jones matrix T as:

$$T = \begin{bmatrix} \text{e}^{\text{i}\cdot F_{\text{LL}}(x,y)} + e^{\text{i}\cdot F_{\text{LR}}(x,y)} + e^{\text{i}\cdot F_{\text{RL}}(x,y)} + e^{\text{i}\cdot F_{\text{RR}}(x,y)} & -\text{i}\cdot(e^{\text{i}\cdot F_{\text{LL}}(x,y)} + e^{\text{i}\cdot F_{\text{LR}}(x,y)} - e^{\text{i}\cdot F_{\text{RL}}(x,y)} - e^{\text{i}\cdot F_{\text{RR}}(x,y)}) \\ \text{i}\cdot(e^{\text{i}\cdot F_{\text{LL}}(x,y)} - e^{\text{i}\cdot F_{\text{LR}}(x,y)} + e^{\text{i}\cdot F_{\text{RL}}(x,y)} - e^{\text{i}\cdot F_{\text{RR}}(x,y)}) & e^{\text{i}\cdot F_{\text{LL}}(x,y)} - e^{\text{i}\cdot F_{\text{LR}}(x,y)} - e^{\text{i}\cdot F_{\text{RL}}(x,y)} + e^{\text{i}\cdot F_{\text{RR}}(x,y)} \end{bmatrix} \tag{S10}$$

Noting that the exterior rotation angle θ only produce opposite equally effect in two cross-polarized L-R and R-L channels, so the phase distribution produced by geometric phase modulation can be expressed as $\varphi_{\text{geo}} = 2\theta = \frac{1}{2}[F_{\text{LR}}(x,y) - F_{\text{RL}}(x,y)]$. Based on the corresponding relationship between Eq. (S9) and (S10), the phase distributions for construction of meta-devices can be deduced by:

$$\varphi_{\text{propa}}^{\text{co}} \propto \frac{1}{2}[F_{\text{LL}}(x,y) + F_{\text{RR}}(x,y)] \tag{S11a}$$

$$\varphi_{\text{chiral}}^{\text{co}} \propto \frac{1}{2}[F_{\text{LL}}(x,y) - F_{\text{RR}}(x,y) + \pi] \tag{S11b}$$

$$\varphi_{\text{propa}}^{\text{cross}} \propto \frac{1}{2}[F_{\text{LR}}(x,y) + F_{\text{RL}}(x,y)] \tag{S11c}$$

$$\varphi_{\text{chiral}}^{\text{cross}} \propto \frac{1}{2}\{[F_{\text{LR}}(x,y) - F_{\text{RL}}(x,y) + 2\varphi' + \pi] \tag{S11d}$$

Firstly, the phase distribution provided by propagation phase in co- and cross-polarized channels should satisfy the conditions in Eq. (S11a) and (S11c) simultaneously, where the initial two co-polarized and two cross-polarized channels are totally same. Then, in order to decouple the inherent consistent performance between co-polarized channels, the chirality-assisted phase pattern for co-polarized component should be designed to fit propagation phase distribution as Eq. (S11b), while it is noted that in Eq. (S11d) $\varphi_{\text{chiral}}^{\text{cross}}$ can produce decoupling effect in two cross-polarized fields. The last

step is to apply exterior rotation to achieve independent wavefronts in two cross-polarized channels, where PB phase distribution is set as $\varphi_{\text{geo}} = 2\theta = \frac{1}{2}[F_{\text{LR}}(x, y) - F_{\text{RL}}(x, y)]$. With the requirement of Eq. (S11) for confirming phase distributions $\varphi_{\text{propa}}^{\text{co}}$, $\varphi_{\text{chiral}}^{\text{co}}$, $\varphi_{\text{propa}}^{\text{cross}}$ and $\varphi_{\text{chiral}}^{\text{cross}}$, each meta-atom at specific location can be designed and optimized to fit synthesized phase modulation, and desired meta-devices can be constructed.

Supplementary Note 4: Establishment of meta-atom library for measurface construction

In order to select the meta-atom to impose the preset phase distributions in all four CP conversion channels simultaneously, the library of meta-atoms is established with independent output phase profiles, as shown in Supplementary Figure 3. Based on the meta-atom library, the specific structure of meta-atom can be immediately obtained when the required four output phase profiles are fixed. It can be observed that with the specific interior angle ϑ, the output co- and cross-polarized phase profiles are functions of length p_x and width p_y of patch. When the interior angle changes, the phases in the two co-polarized channels exhibit distinct tendencies, while the phase response in the two cross-polarized channels are still kept similar, indicating that interior rotation can just decouple the inherent coherence between the two co-polarized outputs under LHCP and RHCP incidences.

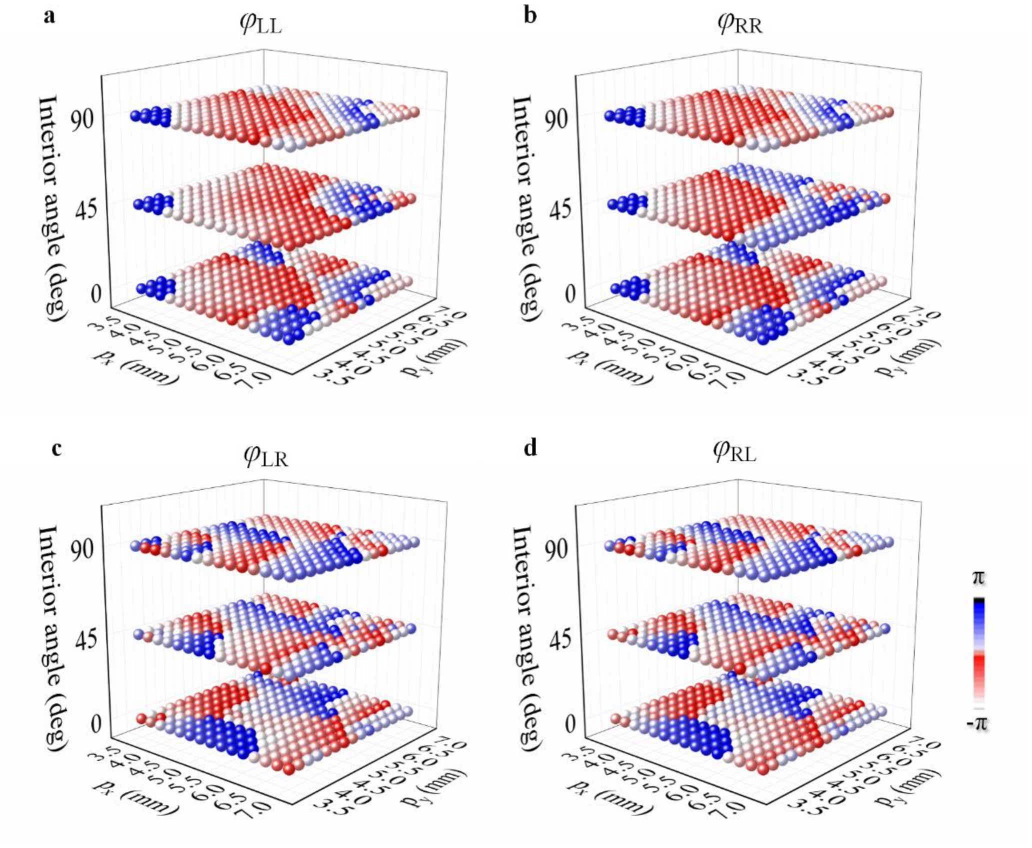

Supplementary Figure 3. Meta-atom library with parameter sweeping of length p_x, width p_y and interior rotation angle ϑ. **a** Simulated co-polarized output phases and **c** cross-polarized output phases with LHCP incidence. **b** Simulated co-polarized output phases and **d** cross-polarized output phases with RHCP incidence. The solid sphere presents the different geometric sizes of patch layer and interior rotation angle, while the color shows the corresponding phase result.

Supplementary Note 5: Discussion on transmission amplitude of meta-atoms

In this part, the amplitude response of meta-atoms is discussed. Here, we apply four parameter degrees of freedom to manipulate all phase patterns of four transmission coefficients independently and simultaneously. The dimension sizes of patch layer (p_x and p_y) can originally set the phase profile of diagonal and off-diagonal elements, achieving independent φ_{LL} (= φ_{RR}) and φ_{LR} (= φ_{RL}). Then the interior angle ϑ can produce chiral responses in structure, which can decouple the phase response between φ_{LL} and φ_{RR}. After that, the exterior angle θ is applied to impose equal and opposite phase interruption in two cross-polarized components, resulting in the distinct response of φ_{LR} and φ_{RL}. These four degrees of freedom for decoupling the inherent consistency between four phase responses are the primary conditions within the whole phase modulation scheme.

It is noted that when there is no interior or exterior rotation ($\vartheta = \theta = 0$), the amplitude ratio between the diagonal and off-diagonal components can be determined by the phase difference between the linearly polarized phase responses along the fast- and slow-axes of birefringent meta-atom as illustrated in Eq. (S4), which is guaranteed by p_x and p_y. Therefore, if the amplitudes are modulated by this linear-phase-difference factor, p_x and p_y would be fixed and limited by this condition. This means these two parameter degrees of freedom to define original phase pattern of diagonal and off-diagonal components would be invalid. Considering the primary condition for our proposed synthesis of three kinds of phase resources, phase modulation is more important than amplitude manipulation. Thus, the amplitude is indeed not considered and partly sacrificed during the optimization process to guarantee the phase requirements.

As mentioned in the main text, there are 25 meta-atoms collected to provide the required phase gradients in all four channels along *x*-direction. The calculated and simulated phase profiles of all meta-atoms are exhibited in Fig. 2b in the main text, while the corresponding simulated output amplitudes of CP conversion channels L-L. L-R, R-L, and R-R are illustrated in Supplementary Figure 4. It can be further observed that the amplitude responses of meta-atoms are not homogeneous, which is scarified to the phase limitations as explained above. We have to say that the non-uniform amplitudes would have some influence in energy distribution, such as the intensity mappings shown in Fig. 5a and 5b, where the doughnut-shape energy rings of vortex beams exhibit some heterogeneity. However, these unequal amplitude responses would not affect the phase distribution，meaning that the proposed phase-modulation scheme would not be limited by the sacrificed amplitude responses.

For future works, an extra degree of freedom needs to be explored to modulate the amplitude profiles so as to achieve independent manipulation of phase and energy in the four CP transmission channels.

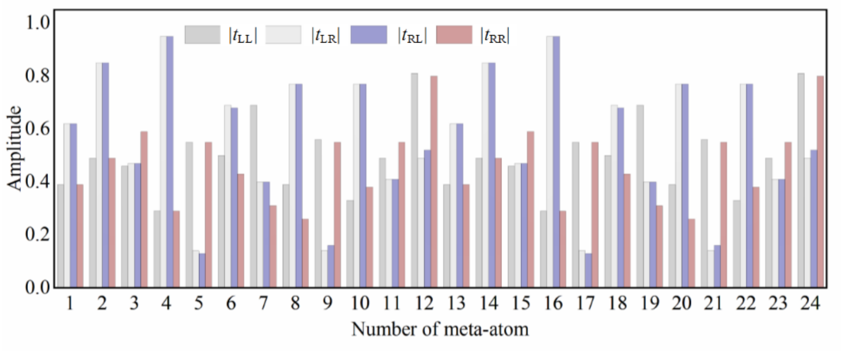

Supplementary Figure 4. Simulated amplitudes of collected 25 meta-atoms for construction of proposed meta-deflector.

Supplementary Note 6: Discussion on spin-to-orbital angular momentum meta-convertor

The non-uniform ring-shaped energy distribution is attributed to the inhomogeneous transmission amplitudes of all the 24 meta-atoms applied to construct the meta-device, as discussed in Supplementary Note 5. Additionally, the step size of the field mapping measurement is ten times as large as the mesh used in the FDTD simulation, so the number of pixels in the measured intensity and phase maps are only 1% of that in the simulated maps. This makes the measurement results seem a little bit rough compared with the simulation results. However, all the above results of spin-to-orbital angular momentum meta-convertor convincingly prove the feasibility of proposed arbitrary manipulation of full spin conversion channels. This single multi-functional meta-converter can simultaneously motivate four different OAM modes just by adjusting the spin states of transmitting and receiving ends, providing a higher plateau for designing functionality integration and equipment miniaturization in modern communication systems.

Supplementary Note 7: Measurement system in microwave region.

The schematic representation of the measurement system for near-field mappings and far-field patterns are exhibited in Supplementary Figure 5, and a brief introduction of experimental setups are illustrated in the main text. Here we will discuss about the processing method of measured data.

In the microwave experimental systems, the transmitting and receiver ends are both connected to the network analyzer, which is adopted to measure the complex S_{11} and S_{21} parameters including the amplitude and phase information.

Here S_{11} and S_{21} parameters represent the reflection and transmission coefficients of the system under test, respectively. The probe is oriented in two directions in order to measure the two components $\vec{E}_{\mathrm{x}}$ and $\vec{E}_{\mathrm{y}}$ (horizontal and vertical) of the transmitted electric field, and then the CP transmitted field at one fixed pixel can be calculated. For the LHCP incidence, co-polarized component can be expressed by $\vec{E}_{\mathrm{co}} = \vec{E}_{\mathrm{x}} + i \cdot \vec{E}_{\mathrm{y}}$, and $\vec{E}_{\mathrm{cross}} = \vec{E}_{\mathrm{x}} - i \cdot \vec{E}_{\mathrm{y}}$ for cross-polarized component, including both amplitude and phase. When flipping the circular polarization state from LHCP and RHCP of the incident horn antenna, the output components in all four CP conversion channels can then be obtained. With the variation of the position of the field probe via the motion controller, the *xoy* and *xoz* planes can be totally covered, and the experimental near-field intensities and phase profiles can be scanned.

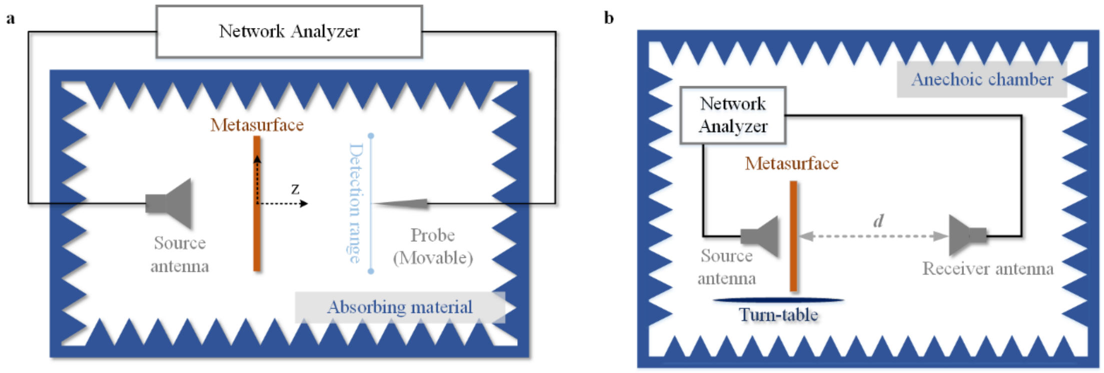

Supplementary Figure 5. Schematic illustration of the measurement setup for **a** near-field mapping and **b** far-field pattern.

Supplementary Note 8: Demonstration of metasurface with arbitrary refraction angles in four CP channels.

In order to verify the independence between functionalities of different CP channels, we constructed another meta-deflector which can refract output wavefronts into different directions with $\varsigma_1^{\text{out}} = 16°$ ($\Delta\Phi_1 = -\pi/6$) for L-L channel, $\varsigma_2^{\text{out}} = -25°$ ($\Delta\Phi_2 = \pi/4$) for L-R channel, $\varsigma_3^{\text{out}} = -8°$ ($\Delta\Phi_3 = \pi/12$) for R-L channel, and $\varsigma_4^{\text{out}} = -58°$ ($\Delta\Phi_4 = \pi/2$) for R-R channel. With the normal illumination of orthogonal CP incidence respectively, the simulated far-field patterns at 10 GHz are shown in Supplementary Figure 6, where the simulated peaks of four far-field patterns are located at required deflection angles, agreeing well with the preset arbitrary conditions for four CP channels.

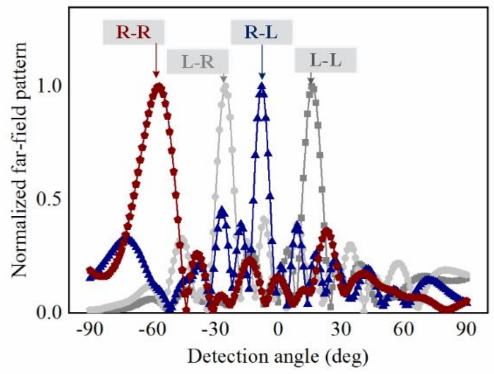

Supplementary Figure 6. Normalized far-field intensities of proposed meta-deflector for verifying the arbitrary tilting angles of different CP channels.
